# Pharmacological Characterization of Toludesvenlafaxine as a Triple Reuptake Inhibitor

**DOI:** 10.3389/fphar.2021.741794

**Published:** 2021-09-14

**Authors:** Haibo Zhu, Wenyan Wang, Chunjie Sha, Wei Guo, Chunmei Li, Fengjuan Zhao, Hongbo Wang, Wanglin Jiang, Jingwei Tian

**Affiliations:** ^1^ School of Pharmacy, Key Laboratory of Molecular Pharmacology and Drug Evaluation, Ministry of Education, Collaborative Innovation Center of Advanced Drug Delivery System and Biotech Drugs in Universities of Shandong, Yantai University, Yantai, China; ^2^ State Key Laboratory of Long-acting and Targeting Drug Delivery System, R&D of Luye Pharmaceutical Group, Yantai, China

**Keywords:** toludesvenlafaxine, triple reuptake inhibitor, antidepressant, pharmacokinetics, rats

## Abstract

Toludesvenlafaxine hydrochloride dihydrate is a novel chemical entity and a potential triple monoamine reuptake inhibitor. This study characterized the *in vitro* triple reuptake inhibition activity, antidepressant-like activity in animals, and pharmacokinetic profiles in rats of toludesvenlafaxine. Binding affinity was determined using human serotonin transporter (SERT) protein, norepinephrine transporter (NET) protein and dopamine transporter (DAT) protein, and the reuptake inhibition was determined using Chinese hamster ovary cells expressing human SERT, NET and DAT. The antidepressant-like activity was examined in rat chronic unpredictable mild stress model and olfactory bulbectomized model. In rats, the tissue distribution and pharmacokinetic parameters were determined. Toludesvenlafaxine had high binding affinity on SERT, NET and DAT, and significantly inhibited the reuptake of serotonin (IC_50_ = 31.4 ± 0.4 nM), norepinephrine (IC_50_ = 586.7 ± 83.6 nM) and dopamine (IC_50_ = 733.2 ± 10.3 nM) *in vitro*. Toludesvenlafaxine demonstrated significant antidepressant-like effects in rat models at 8–16 mg/kg. In addition, toludesvenlafaxine significantly reduced serum corticosterone and significantly increased testosterone levels in rats. Toludesvenlafaxine was quickly absorbed and converted to *O*-desvenlafaxine (ODV) after oral administration, both of which were selectively distributed into the hypothalamus with high concentration. Plasma ODV exposure was proportionally related to the doses after oral dosing. These results suggest that toludesvenlafaxine is a triple reuptake inhibitor with relatively fast-acting antidepressant-like activity and good therapeutic profile including improvement of anhedonia and sexual function.

## Introduction

More than 350 million people across the globe suffer from major depressive disorder (MDD), which is a debilitating and refractory disease that is ranked second globally in terms of disease burden (Smith, 2014). Depression has become a serious global public health challenge due to its characteristics of high morbidity incidence, high suicidal rate, high recurrence rate and high disability rate (Otte et al., 2016). Several different classes of antidepressants have been used to treat depression such as tricyclic antidepressants (TCAs), selective serotonin (5-HT) reuptake inhibitors (SSRIs), and serotonin-norepinephrine (NE) reuptake inhibitors (SNRIs). In general, currently available antidepressants are effective in the treatment of depression but most have various shortcomings such as delayed onset of action, limited therapeutic efficacy with modest response rate (50–70%), lack of cognitive improvement, causing sexual dysfunction and suicidal tendencies (Dupuy et al., 2011). For example, venlafaxine was the first marketed SNRI antidepressant and its main active metabolite, desvenlafaxine (*O*-desmethylvenlafaxine, ODV; its succinate monohydrate salt [DVS] is marketed as Pristiq®) which is also a SNRI, was later marketed as an improved antidepressant than its parent compound, but both still have significant unwanted effects such as gastrointestinal discomfort and sexual dysfunction (Shelton, 2019). Therefore, safer and effective antidepressants are in high demand to meet the clinical needs.

In recent years, the triple monoamine reuptake inhibition hypothesis has been increasingly recognized as a potentially beneficial strategy to treat depression (Subbaiah, 2018). Dopamine (DA) plays an essential role in the etiology of depression (Dunlop BW and Nemeroff, 2007; Belujon and Grace, 2017). Increasing the synaptic dopamine level in the mesolimbic cortex can improve the late onset effect of antidepressants. Elevation of the dopamine level in the hypothalamus enhances the dopaminergic neural tone in the nucleus accumbens, which can improve anhedonia, cognitive function, rewarding motivation and goal-directed behaviors, and reduce sexual dysfunction related to the use of SSRIs and SNRIs (Belujon and Grace, 2017). For example, bupropion is a weak inhibitor of DA and NE transporters and, when combined with SSRIs or SNRIs, it can shorten the onset of antidepressant action and reduce the occurrence of sexual dysfunction (Trivedi et al., 2006; Zisook et al., 2006). In addition, the SSRI citalopram has been found to significantly improve the onset of action when combined with the dopaminergic agent methylphenidate (Lavretsky et al., 2006). Combined, these results suggest that triple reuptake inhibitors which can enhance the functionality of all three monoamines may have improved therapeutic profile to treat depression such as faster onset, improved cognitive function, less anhedonia and sexual dysfunction (Prins et al., 2011).

**FIGURE 1:**
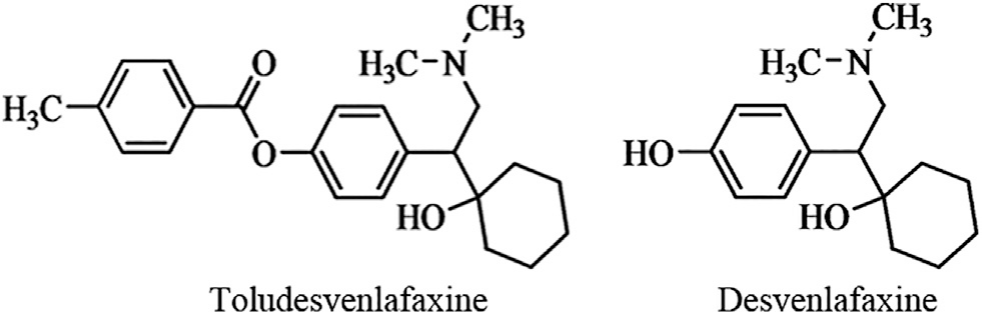
The chemical structure of toludesvenlafaxine and desvenlafaxine.

Toludesvenlafaxine hydrochloride ((±)-4-(2-(dimethylamino)-1-(1-hydroxycyclohexyl) ethyl) phenyl 4-methylbenzoate hydrochloride) (also known as LPM570065 or ansofaxine) is a new chemical entity and a potential triple reuptake inhibitor (Figure 1). It is a prodrug that can quickly converts to the SNRI desvenlafaxine under the hydrolysis of ubiquitous esterase *in vivo* (Zhang et al., 2014). Microdialysis study shows that toludesvenlafaxine can increase the serotonin (5-HT), norepinephrine (NE) and dopamine (DA) levels in the striatum after oral drug administration while, as expected, desvenlafaxine does not increase the striatal DA level (Zhang et al., 2014). These results suggest that toludesvenlafaxine may have unique pharmacological effects beyond its conversion to desvenlafaxine. The goals of the current study were three-fold: 1) despite the neurochemical evidence that toludesvenlafaxine was able to increase the central 5-HT, NE and DA levels (Zhang et al., 2014), conclusive *in vitro* binding and functional data supporting toludesvenlafaxine as a triple reuptake inhibitor have not been reported, which will be examined in this study; 2) although toludesvenlafaxine has been shown to reduce the immobility time in the forced swim test (Zhang et al., 2014), forced swim test as a rodent model of depression has been controversial and it is more appropriate to be used as a quick screening assay for potential antidepressant-like activities (Nestler and Hyman, 2010); as such, this study further evaluated the antidepressant-like activity of toludesvenlafaxine using two rodent models of depression with higher validity: chronic unpredictable mild stress model and olfactory bulbectomized model; 3) although toludesvenlafaxine has been shown to be able to enter the brain after oral and intravenous administration (Zhang et al., 2014), detailed pharmacokinetic profile remains unknown, which will be examined in this study.

## Materials and Methods

### Chemicals and Reagents

Toludesvenlafaxine hydrochloride and desvenlafaxine succinate monohydrate (DVS) were provided by State Key Laboratory of Long-acting and Targeting Drug Delivery Technologies (Yantai, PR China). The compound purities used in the present study were examined by HPLC and all were higher than 99%. For the *in vitro* study, toludesvenlafaxine was dissolved in dimethyl sulfoxide (DMSO) and for *in vivo* studies, toludesvenlafaxine and DVS were suspended with 0.5% sodium carboxymethylcellulose (SCMC) at the proposed doses. Cell lines expressing human DAT, NET and SERT were provided by PerkinElmer Life and Analytical Sciences (United States). 5-HT, NE and DA were all purchased from Sigma (St. Louis, MO, United States). DOV21947 (also known as amitifadine, a triple reuptake inhibitor) was provided by Shanghai Institute of Pharmaceutical Industry (Shanghai, China). Corticosterone and testosterone test kits were obtained from EMD Chemicals Inc. (San Diego, CA, United States).

### Cell Lines and Animals

Male and female Wistar rats (200–220 g) used for pharmacokinetics study and tissue distribution study were provided by the Animal Research Institute, Jilin University. Male and Female Sprague-Dawley (SD) rats (180–220 g) used for pharmacodynamics studies were provided by Shandong Luye Pharmaceutical Co. Ltd. All animals were housed in a light- and temperature-controlled room (12-h light/dark cycle, lights on at 06:00 h and off at 18:00 h, 21–22°C, humidity 60–65%) and maintained on a standard diet with continuous access to water. All procedures related to animal experiments in the study complied with the relevant laws and regulations in the experimental animal use and management and the relevant regulations in Institutional Animal Care and Use Committee (IACUC) in our management agency. The number of animals, experimental design and animal handling were approved by IACUC and were implemented strictly in accordance with the protocols.

### *In Vitro* Binding and Reuptake Inhibition in Cells Expressing Human 5-HT, NE and DA Transporters

Affinity of toludesvenlafaxine to DA, NE, and 5-HT transporters was determined by radioligand membrane binding assay according to published protocol (Deecher et al., 2006). Methods and procedures of this study complied with the standard operating procedures. The inhibition ratio was calculated from “100-binding ratio.” Binding was calculated by the equation ([(B-N)/(B_0_-N)]∗100 (%)) and the dose-response curves and K_i_ values were calculated.

Reuptake inhibition study was conducted using procedures identical to those described by Eshleman et al. (1999). Chinese hamster ovary (CHO) cells that stably express DAT, NET or SERT were grown on 150 mm-diameter tissue culture dishes. Aliquots (50 μL) of the suspended cells were added to assay tubes (in triplicate) containing DOV21947 or toludesvenlafaxine and Krebs–HEPES assay buffer in a final assay volume of 0.5 ml. Following a 10-min preincubation in a 25°C water bath, [^3^H] neurotransmitter (final concentration, 20 nM; specific activity of [^3^H] DA, [^3^H] serotonin, and [^3^H] NE: 40–60, 23.7, and 56 Ci/mmol, respectively) was added and incubated for 10 min. The reaction was terminated by filtration through Wallac filtermat A filters, presoaked in 0.05% polyethylenimine, using a 96-well Tomtec cell harvester. Scintillation fluid was added to each filtered spot, and radioactivity remaining on the filters was measured by liquid scintillation spectrometry.

### Plasma, Brain and Hypothalamus Distribution of Toludesvenlafaxine in Rats

Toludesvenlafaxine absorption kinetics in rat plasma was conducted in Wistar rats (12/sex/group) following a single intragastric dose at 4, 8 or 16 mg/kg. Plasma samples were collected at pre-dose, 0.167, 0.33, 0.5, 1, 1.5, 2, 3, 4, 6, 8, 12 and 24 h after dosing for PK analysis. Plasma concentration and PK parameters of the major metabolite of toludesvenlafaxine, *O*-desvenlafaxine (ODV), were determined. The blood samples were centrifuged at 3,500 rpm for 5 min, then the plasma was separated and stored at −80°C until analysis using LC-MS/MS, which includes an Agilent 1,100 Serious HPLC (Agilent Technologies, Palo Alto, CA, United States) coupled to a QTRAP 2000™ mass spectrometer (Applied Biosystems Sciex, United States) equipped with an ESI source.

Total brain and hypothalamus distribution study was conducted in Wistar rats (4/sex/group) following a single intragastric dose at 8 mg/kg. Hypothalamus and brain tissues were collected at 0.25, 1, or 12 h post-dosing. All tissues were immediately rinsed with 200 μg/ml dichlorovos (as esterase inhibitor) ice-cold saline after collection and then were stored at −80°C until analysis using LC-MS/MS.

### Chronic Unpredictable Mild Stress Procedure

The chronic unpredictable mild stress (CUMS) procedure was adapted from published literature (Jason et al., 2013). Briefly, SD rats were randomly exposed to various stressors for 3 weeks. The stressors applied included: deprivation of food or water (17 h) or of both water and food deprivation (22 h), overnight illumination, forced cold swimming (40°C, 5 min), soiled cage (18 h), tail pinch (1 min), white noise (9 h) or 45° cage tilt (18 h). No stressor was repeated within 2 days for each rat. The control group was housed in a separate room, with free access to food and water, and not disturbed.

Five groups (5 rats/sex/group) of SD rats were used (a total of 50 rats with male and female rats equally distributed): control group, CUMS model group, DVS 7.3 mg/kg group (as a comparison drug), and toludesvenlafaxine (4 and 8 mg/kg) groups. Rats received oral administration of study drugs once daily for 21 consecutive days during the chronic stress exposure period. At the end of the chronic stress and drug treatment period, the open field test (21st day) and amount of sucrose consumption (22nd day) were determined.

### Rat Olfactory Bulbectomized Model

The rat olfactory bulbectomized model was adapted from published literature (Morales-Medina et al., 2017). After surgery, the rats were returned to home cages and handled daily for 5–10 min. After recovery from the surgery, the rats were submitted for the open field test and were assigned into five groups based on their baseline locomotion levels to ensure no significant between-group differences: the vehicle group (0.5% SCMC), DVS 7.3 mg/kg group (as a comparison drug) and toludesvenlafaxine 4, 8 and 16 mg/kg groups. Thereafter, all rats received daily intragastric dosing for 20 days. The open field test on the 7th and 14th days of dosing, sucrose consumption on the 18th day of dosing, and serum levels of corticosterone and testosterone on the 20th day of dosing were determined.

### Open Field Test

Open field test was conducted to examine behavioral changes in the rats as altered activity in the open field test was a hallmark change in these models: hyperactivity in rats that received olfactory bulbectomy (Kelly et al., 1997; Machado et al., 2012) and hypoactivity in rats that received CUMS (Hu et al., 2017). Locomotor activity was measured in custom-made acrylic boxes (Height✕Width✕Length: 40✕52✕52 cm). The boxes were placed underneath cameras and all sessions (the duration of the sessions was 5 min) were recorded and data were later quantified by experimenters blinded to treatments. The bottom surface of the boxes was divided into 16 equal squares. Two parameters were quantified from the sessions: the number of squares each rat crossed with both front paws and the number of rearing episodes. Standing with two front paws off the ground was considered one episode of rearing.

### Sucrose Consumption Test

The sucrose intake test is a standard test to evaluate the level of anhedonia to a sweet reward in both models of depression and was conducted according to methods described in a previous study with minor modifications (Jang et al., 2004). Rats were exposed to 1% sucrose solution in leak-proof bottles for 1 h per day for 3 consecutive days. The test was carried out after 22 h of food and water deprivation. The weight difference of the bottles before and after the 2-h test was considered the total amount of sucrose consumed by the rats.

### Measurement of the Corticosterone and Testosterone Levels

The serum corticosterone and testosterone levels were measured following the kit instructions.

### Statistics

Data were expressed as mean ± standard deviation (STD). Multigroup comparisons of the means were carried out by one-way analysis of variance (ANOVA) test with post hoc contrasts by Dunnett’s test. *p* < 0.05 was considered significant.

## Results

### *In Vitro* Binding Affinities of Toludesvenlafaxine

Toludesvenlafaxine at 10 μM showed high binding affinities to SERT, NET and DAT. The 50% inhibiting concentration (IC_50_) values of toludesvenlafaxine on radioligand binding to SERT, NET and DAT were 1,330 ± 82.5 nM, 2,200 ± 278 nM, and 227 ± 21.7 nM, respectively, and the corresponding K_i_ values were 763 ± 48.7 nM, 2040 ± 258 nM and 197 ± 18.7 nM, respectively.

### *In Vitro* Reuptake Inhibition Effects of Toludesvenlafaxine on Dopamine, Norepinephrine, and 5-Hydroxytryptamine

The inhibition of toludesvenlafaxine on the [^3^H] neurotransmitter reuptake was determined by radioligand assay in CHO cells expressing human SERT, NET and DAT. The IC_50_ values for 5-HT, NE and DA reuptake were 31.4 ± 0.4 nM, 586.7 ± 84 nM, and 733.2 ± 10 nM, respectively, as shown in Table 1. As a comparison, the reuptake-inhibiting effects of DVS on NE and 5-HT were also tested which were similar to toludesvenlafaxine (IC_50_ values for 5-HT and NE reuptake were 53.46 ± 7.55 nM, and 568.7 ± 29.85 nM of DVS, respectively). However, DVS demonstrated no inhibition on DA reuptake, which was expected but was different from toludesvenlafaxine. The known triple reuptake inhibitor DOV21947 showed clear inhibition on the reuptake of all the three monoamine neurotransmitters (The IC_50_ values for 5-HT, NE and DA reuptake were 78.58 ± 16.19 nM, 223.1 ± 97.98 nM, and 197.15 ± 25.8 nM of DOV21947, respectively) (Table 1).

**TABLE 1:** Inhibitory effects and IC_50_ values of toludesvenlafaxine on 5-HT, NE and DA reuptake.

Compound	IC_50_ (nM)		
	DA	NE	5-HT
Toludesvenlafaxine	733.2 ± 10.3	586.7 ± 83.6	31.4 ± 0.4
DOV21947 ^a^	197.1 ± 25.8	223.1 ± 98.0	78.6 ± 16.2
DVS	-	568.7 ± 29.9	53.5 ± 7.6

- = not calculated; 5-HT = 5-hydroxytryptamine; DA = dopamine; DVS = *O*-desvenlafaxine succinate; NE = norepinephrine.

The inhibition of toludesvenlafaxine hydrochloride on the [^3^H] neurotransmitter (5-HT, NE and DA) reuptake was determined by radioligand assay in CHO cells expressing human SERT, NET or DAT, and each assay was repeated 3 times. Toludesvenlafaxine hydrochloride and DOV21947 (positive control) at concentration ranges from 1 × 10^–4^–10 μM were incubated individually with CHO cells expressing different transporters, followed by adding corresponding radiolabeled neurotransmitters. 50% inhibition of the reuptake (IC_50_) of toludesvenlafaxine hydrochloride on human 5-HT, NE and DA was calculated. Values were presented as mean ± S.E. (*n* = 3).

a = DOV21947, also known as amitifadine or EB-1010.

### Effects of Toludesvenlafaxine on CUMS in Rats

As shown in Table 2, the CUMS model rats demonstrated significantly lower locomotion in the open field test with both number of squares crossed and rearing episodes significantly lower than the control group (F_(4, 45)_ = 5.482, *p* < 0.01 compared to control group). In addition, the CUMS rats consumed significantly less sugar solution during test period compared to the control group (F_(4, 45)_ = 14.078, *p* < 0.01 compared to control group), suggesting that the rats that experienced CUMS demonstrated clear depression-like behaviors such as hypoactivity and anhedonia. Chronic toludesvenlafaxine treatment at the dose of 8 mg/kg significantly prevented CUMS-induced hypoactivity (F_(4, 45)_ = 5.482, *p* < 0.01 compared to vehicle group) in the open field test. In addition, toludesvenlafaxine at the doses of 4 and 8 mg/kg significantly prevented the reduced rearing frequency (F_(4, 45)_ = 14.078, *p* < 0.01 compared to vehicle group) and the reduction of sucrose consumption (F_(4, 45)_ = 5.482, *p* < 0.01 compared to vehicle group) in the CUMS rats. These results suggest that chronic toludesvenlafaxine treatment reduced depressive-like behaviors in the CUMS model. As a comparison, chronic DVS treatment at 7.3 mg/kg (equimolar dose of toludesvenlafaxine 8 mg/kg) also significantly prevented all the three behavioral endpoints and the effect magnitude falls between 4 mg/kg and 8 mg/kg toludesvenlafaxine treatments (Table 2).

**TABLE 2:** Effects of toludesvenlafaxine on the Locomotor activity and sugar consumption in CUMS rats.

Group	Dose (mg/kg)	Sugar consumption rate (%)	Rearing time (seconds)	Numbers of squares crossed
Control^a^	-	89.9 ± 6.15	24.4 ± 6.5	72.3 ± 21.4
Vehicle^b^	-	60.6 ± 13.9^c^	6.8 ± 3.3^c^	29.7 ± 13.9^c^
DVS^b^	7.3	82.1 ± 7.4^d^	15.2 ± 3.9^d^	47.0 ± 12.1^e^
Toludesvenlafaxine^b^	4	80.0 ± 7.7^d^	13.6 ± 6.4^e^	44.3 ± 8.6
Toludesvenlafaxine^b^	8	86.4 ± 3.7^d^	18.6 ± 5.7^d^	53.6 ± 14.0^d^

CUMS = chronic unpredictable mild stress; DVS = *O*-desvenlafaxine succinate.

a = animals not treated with daily stress condition.

b = animals treated with daily stress condition.

c *p* < 0.01 versus control group.

e *p* < 0.05.

d *p* < 0.01 versus vehicle group. Significance was determined by one-way ANOVA followed by Dunnett’s test. Values were presented as mean ± STD (*n* = 10).

### Effects of Toludesvenlafaxine in Olfactory Bulbectomized Rats

The open field test was conducted on the 7th and 14th days of dosing. In olfactory bulbectomized rats, the locomotion and rearing time were both significantly higher in both tests as compared to vehicle-treated rats (Table 3), demonstrating typical hyperactivity in this model. Toludesvenlafaxine treatment at 4–16 mg/kg significantly and dose-dependently reduced the hyperactivity on the 7th day of dosing (F_(5_, _54)_ = 16.901, *p* < 0.01or *p* < 0.05, compared with vehicle group) and 8–16 mg/kg on the 14th day of dosing (F_(5_, _54)_ = 9.21, *p* < 0.01, compared with vehicle group). The time of rearing was also significantly reduced in toludesvenlafaxine treatment groups at 4–16 mg/kg on the 7th (F_(5_, _54)_ = 12.249, *p* < 0.01, compared with vehicle group) and 14th day of dosing (F_(5_, _54)_ = 14.088, *p* < 0.01, compared with vehicle group). Although toludesvenlafaxine reduced both behavioral outputs to the level similar to sham-operated rats, 7.3 mg/kg DVS treatment only significantly reduced the rearing time in these rats and the effect was much smaller than that of toludesvenlafaxine (Table 3).

**TABLE 3:** Effects of toludesvenlafaxine on the Locomotor activity in olfactory bulbectomized rats.

Group	Dose (mg/kg)	Moving distance (squares crossed)		Rearing time (seconds)	
		Day 7	Day 14	Day 7	Day 14
Sham-operated ^a^	-	10.9 ± 3.0	14.7 ± 5.8	6.5 ± 2.5	7.4 ± 2.7
Vehicle ^b^	-	29.3 ± 7.9^c^	27.8 ± 7.4^c^	15.7 ± 7.5^c^	13.8 ± 3.7^c^
DVS ^b^	7.3	26.8 ± 7.6	26.7 ± 10.1	7.0 ± 3.1^d^	9.4 ± 3.1^d^
Toludesvenlafaxine ^b^	4	22.6 ± 8.1^e^	22.1 ± 8.8	5.1 ± 2.2^d^	6.9 ± 2.0^d^
Toludesvenlafaxine ^b^	8	16.8 ± 4.2^d^	15.6 ± 4.8^d^	4.8 ± 2.0^d^	5.0 ± 2.2^d^
Toludesvenlafaxine ^b^	16	10.6 ± 3.8^d^	11.3 ± 3.5^d^	4.2 ± 3.1^d^	5.1 ± 2.8^d^

DVS = *O*-desvenlafaxine succinate.

a = no olfactory bulbectomy.

b = olfactory bulbectomy.

c *p* < 0.01 versus sham-operated group.

e *p* < 0.05.

d *p* < 0.01 versus vehicle group. Significance was determined by one-way ANOVA followed by Dunnett’s test. Values were presented as mean ± STD (*n* = 10).

When tested on the 18th day, the amount of sucrose consumed was significantly reduced in olfactory bulbectomized rats as compared to sham-operated rats, suggesting significant anhedonia (Table 4). Toludesvenlafaxine treatment dose-dependently and significantly increased the amount of sucrose consumption at doses of 8 and16 mg/kg (F_(5_, _54)_ = 2.229, *p* < 0.01 or *p* < 0.05, compared with vehicle group). In contrast, although 7.3 mg/kg DVS also slightly increased the sugar consumption, the change did not reach statistical significance (Table 4). When tested on the 20th day, the serum corticosterone level was found higher in model rats as compared to the sham-operated rats, although the difference did not achieve statistical significance. Toludesvenlafaxine treatment at doses of 4–16 mg/kg dose-dependently and significantly reduced the serum corticosterone level as compared to the model group (F_(5_, _53)_ = 5.477, *p* < 0.01 or *p* < 0.05). In contrast, 7.3 mg/kg DVS treatment did not affect the serum corticosterone level. Interestingly, although the testosterone level was lower in olfactory bulbectomized rats as compared to the sham-operated rats, toludesvenlafaxine tended to dose-dependently increase the testosterone level and at 16 mg/kg achieved statistical significance (F_(5_, _53)_ = 2.76, *p* < 0.05) (Table 4). Again, 7.3 mg/kg DVS treatment did not have significant effect on the testosterone level.

**TABLE 4:** Effects of toludesvenlafaxine on sugar consumption, serum corticosterone and testosterone levels in olfactory bulbectomized rats.

Group	Dose (mg/kg)	Sugar consumption rate (%)	Corticosterone (ng/L)	Testosterone (ng/mL)
Sham-operated ^a^	-	75.8 ± 12.7	407.4 ± 216.5	4.7 ± 3.5
Vehicle ^b^	-	49.7 ± 18.9^c^	502.5 ± 102.0	1.7 ± 1.3
DVS ^b^	7.3	58.1 ± 26.6	394.1 ± 231.6	2.4 ± 1.8
Toludesvenlafaxine ^b^	4	62.1 ± 15.5	281.3 ± 118.8^d^	2.0 ± 1.4
Toludesvenlafaxine ^b^	8	70.7 ± 15.6^d^	206.6 ± 91.2^e^	3.3 ± 2.6
Toludesvenlafaxine ^b^	16	73.4 ± 15.6^e^	222.0 ± 99.6^e^	5.4 ± 4.6^d^

DVS = *O*-desvenlafaxine succinate.

a = no olfactory bulbectomy.

b = olfactory bulbectomy.

c *p* < 0.01 versus sham-operated group.

d *p* < 0.05.

e *p* < 0.01 versus vehicle group. Significance was determined by one-way ANOVA followed by Dunnett’s test. Values were presented as mean ± STD (n = 10).

### Blood, Brain and Hypothalamus Distribution of Toludesvenlafaxine in Rats

Although it is known that toludesvenlafaxine can enter the brain and is detectable in the striatum after oral dosing (Zhang et al., 2014), the pharmacokinetic profile was unknown. After intragastric administration with 8 mg/kg toludesvenlafaxine, very low level of toludesvenlafaxine (0.55 ng/ml) was detected in the blood serum 15 min post-dosing and throughout the study. In contrast, highest level of its major metabolite, ODV, was detected in the blood (90.89 ng/g tissue) 15 min post-dosing, which remained at very high level 1 h post-dosing and largely eliminated 12 h post-dosing (Table 5). These results were consistent with the notion that toludesvenlafaxine as a pro-drug was quickly metabolized into its major active metabolite, ODV, after the drug was absorbed into the blood. Interestingly, although low level of toludesvenlafaxine and moderate level of ODV were detected in the total brain tissue up to 12 h post-dosing, high level of toludesvenlafaxine (68.6 ng/g tissue) and even higher level of ODV (92.52 ng/g tissue) were detected in the hypothalamus 15 min post-dosing which were gradually eliminated 12 h post-dosing (Table 5). These results suggest that after absorption, portion of toludesvenlafaxine was not metabolized but instead quickly re-distributed to the brain and particularly hypothalamus. Hypothalamus also appears to be the brain region with high level of ODV distribution.

**TABLE 5:** Blood, brain and hypothalamus tissue distribution of toludesvenlafaxine and ODV following *intragastric* administration of Toludesvenlafaxine at 8 mg/kg in Rats.

	Time	Brain (ng/g)	Hypothalamus (ng/g)	Blood (ng/mL)
Toludesvenlafaxine	15 min	4.00 ± 4.38	68.6 ± 77.45	0.55 ± 0.48 ^b^
	1 h	1.45 ± 1.01	46.12 ± 41.44	0.34 ± 0.47
	12 h	0.41 ± 1.17	2.56 ± 4.98	0.09 ± 0.26
ODV	15 min	13.78 ± 8.40	92.52 ± 68.23	90.89 ± 33.55 ^b^
	1 h	20.88 ± 6.80	97.88 ± 57.98	68.20 ± 23.56
	12 h	2.06 ± 1.29	7.22 ± 6.79	2.84 ± 2.69

a = hours; ODV = *O*-desmethyl venlafaxine.

ODV, the active metabolite of toludesvenlafaxine, following oral administration of toludesvenlafaxine at 8 mg/kg in rats.

b the concentration was detected at 20 min after Toludesvenlafaxine administration by oral.

Ten min after intragastric administration with 8 mg/kg toludesvenlafaxine, ODV was detected in the rat plasma, with T_max_ being 0.48 ± 0.23 h and C_max_ being 101.23 ± 30.99 ng/ml (Table 6). The three doses of toludesvenlafaxine showed similar T_max_, and the C_max_ and AUC_0-∞_ were dose-dependent (Table 6).

**TABLE 6:** Pharmacokinetic parameters of ODV after single administration of toludesvenlafaxine in rats.

Dose (mg/kg	T_max_ (h)	C_max_ (ng/mL)	AUC_0-t_ (ng·h/mL)	AUC_0-∞_ (ng·h/mL)	C_L_/F (L/h/kg)	t_1/2_ (h)
4	0.52 ± 0.20	38.88 ± 21.86	82.13 ± 54.95	89.29 ± 54.45	61.25 ± 35.00	3.79 ± 1.12
8	0.48 ± 0.23	101.23 ± 30.99	241.07 ± 101.26	251.15 ± 109.95	36.44 ± 12.38	2.49 ± 0.44
16	0.60 ± 0.25	219.13 ± 59.65	567.95 ± 189.70	606.69 ± 196.41	29.33 ± 11.13	3.49 ± 1.11

## Discussion

The primary findings of the current study were that toludesvenlafaxine demonstrated characteristic tri-reuptake inhibitor profile in *vitro* assays, which showed highest inhibition activity at SERT. In preclinical rat models of depression, toludesvenlafaxine demonstrated dose-dependent and significant antidepressant-like activity. In preclinical pharmacokinetic study, toludesvenlafaxine was well-absorbed into the brain with highest drug concentration detected at hypothalamus. Overall, these results reveal toludesvenlafaxine as a tri-uptake inhibitor and has the potential to act as a safe antidepressant agent.

One major issue with currently wide prescribed antidepressants including SSRIs and SNRIs is the slow onset of action and limited therapeutic efficacy. Many patients with MDD only partially respond to and some have no clinically meaningful response to these antidepressants. In addition, potent 5-HT reuptake inhibition often leads to hypodopaminergic adverse effects such as decreased sexual function, weight gain and emotion blunt (Nutt, 2008). Mesolimbic dopaminergic system is crucially involved in cognition, motivation and reward-related behaviors (Nestler and Carlezon, 2006). Therefore, the inclusion of DA reuptake inhibition may elevate the mesolimbic dopaminergic activity and ameliorate some 5-HT reuptake inhibition-related adverse effects, which is a primary rationale for the proposed tri-reuptake inhibitors as potential treatments of MDD. Clinical evidence that the combination therapy with the DA transporter blocker bupropion and SSRIs is effective in SSRI-resistant MDD patients (Zisook et al., 2006) seems in support of this notion. Over the years, several TRIs with varying potencies in inhibiting monoamine transporters have been developed and tested in preclinical and clinical studies with some success and interest remains strong in developing TRIs for the treatment of MDD (Sharma et al., 2015).

In this study, toludesvenlafaxine demonstrated high affinities at SERT, NET and DAT and differential inhibition potencies at the three transporters in cell-based *in vitro* assays, with the binding affinities ratio of 2.7: 1: 10.4, respectively. The reuptake inhibition ratio of toludesvenlafaxine at 5-HT, NE and DA was 23.3: 1.2: 1, showing clear preference for inhibiting 5-HT reuptake. As a comparison, DVS only shows reuptake inhibition for 5-HT and NE (IC_50_ values were 53.5 ± 7.6 nM and 568.7 ± 29.9 nM, respectively), with an inhibition ratio of 10.6:1, similar to that of toludesvenlafaxine (Table.1). However, 3.9-fold higher affinity of toludesvenlafaxine to DAT compared to SERT, whereas 23.3-fold higher reuptake inhibition of toludesvenlafaxine to 5-HT compared to DAT. This discrepancy may be due to different transporter expression levels, different methodology or in different laboratories (Ilic et al., 2020). These results are consistent with a previous study showing that toludesvenlafaxine increases the striatal 5-HT, DA and NE levels while DVS only increases the striatal 5-HT and NE levels (Zhang et al., 2014). Together, these findings strongly suggest that toludesvenlafaxine is a triple reuptake inhibitor at 5-HT, NE and DA.

CUMS model is a well-validated animal model that mirrors many aspects of human depression symptomology such as anhedonia, decreased motivation and locomotion, and elevated stress hormone level (Willner, 2016). Likewise, olfactory bulbectomized model is well-validated and has decades of research literature to support its utility as a valuable animal model of depression (Harkin et al., 2003; Morales-Medina et al., 2017). In this study, it was found that although both DVS and toludesvenlafaxine significantly prevented hypoactivity and anhedonia in the CUMS rats, toludesvenlafaxine seems to be more effective than DVS. In contrast, DVS was largely ineffective in the rat olfactory bulbectomy model, but chronic treatment with toludesvenlafaxine was able to significantly prevent the hyperactivity, anhedonia and elevated serum corticosterone level (Tables 3 and 4). Together, these results suggest that toludesvenlafaxine shows significant antidepressant-like activity and may be more effective than DVS at similar doses.

Conventional antidepressants such as SSRIs or SNRIs usually need several weeks to exert antidepressant effect, and maximal effect may occurred in 6–8 weeks of treatment (F Artigas et al., 2018). The activation of mesolimbic dopaminergic neurons plays central role in the control of motivation and reward related behaviors that are frequently blunted in depression (Naranjo et al., 2001). Thus, immediate increases in synaptic dopamine levels (via inhibition of dopamine reuptake) may result in a more rapid relief of symptoms associated with anhedonia than produced by SSRIs or SNRIs (Skolnick et al., 2006). Toludesvenlafaxine treatment at 4–16 mg/kg significantly and dose-dependently reduced the hyperactivity on the 7th day of dosing. These results suggest that toludesvenlafaxine may have fast-acting antidepressant-like activity, and are consistent with the previous findings of tri-reuptake inhibitor (Prins et al., 2011).

One common side effect with the use of antidepressants in general, and SSRIs in particular, is sexual dysfunction. Although the exact mechanisms that cause sexual dysfunction remain elusive, it is suggested that the inhibitory effects of serotonergic antidepressants lead to blunted dopamine release in hypothalamic and mesolimbic areas and neuroendocrine imbalance such as elevated prolactin secretion and reduced testosterone level (Lyons et al., 2016; Bijlsma et al., 2014). In this study, while the rats that received olfactory bulbectomy surgery showed marked reduction of serum testosterone level, the difference did not reach statistical significance primarily due to the large variability in the sham-operated rats. Nonetheless, chronic toludesvenlafaxine treatment dose-dependently and significantly prevented the testosterone reduction while DVS showed no significant effect (Table 4). This result is consistent with and may be explained by the high concentration of both toludesvenlafaxine and its primary metabolite ODV in hypothalamus after oral dosing. In our previous studies, repeated treatment with toludesvenlafaxine (30–300 mg/kg) for 63 days dose-dependently increased sperm density and count in male rats for up to 24.1% (Guo et al., 2018). In addition, prolonged treatment with toludesvenlafaxine (30–300 mg/kg) for 91 days dose-dependently reduced the serum prolactin level by as much as 78% and increased the serum testosterone level by up to 37% (Li et al., 2018). Given the strong correlation between low testosterone level and depression symptomology and the effectiveness of testosterone supplement therapy in depressed men (Walther et al., 2019), these results strongly suggest that toludesvenlafaxine may have low risk of leading to sexual dysfunction but instead its effect on androgen level may benefit its antidepressant effect and improve the sexual function in patients with depression, which ultimately will improve the quality of life of these patients.

In an effort to understand the bioavailability and pharmacokinetic profile of oral toludesvenlafaxine administration, rats were used and toludesvenlafaxine was found to be quickly converted into ODV after drug absorption. Only trace amount of toludesvenlafaxine was detected in the rat plasma after 8 mg/kg toludesvenlafaxine administration. The analysis of tissue distribution found that after intragastric toludesvenlafaxine administration, the drug quickly entered the central nervous system and highest concentrations of both toludesvenlafaxine and ODV were found in the hypothalamus at much higher level than in the plasma. This finding suggests that, for reasons currently unclear, portion of toludesvenlafaxine was not converted to ODV after absorption but quickly redistributed to certain tissues including hypothalamus. Although it is currently unclear the implications of this distribution profile in the therapeutic effects of toludesvenlafaxine, the drug and its metabolite ODV were eliminated quite slowly in the hypothalamus, detectable even 12 h after drug dosing. In a separate study using Beagle dogs as experimental subjects, it was found that after oral administration of 80 mg toludesvenlafaxine extended release tablets the drug was quickly converted into ODV and the plasma toludesvenlafaxine level was below detection limit. However, toludesvenlafaxine was detectable in the dog hypothalamus and the whole brain tissue (unpublished data). Together, these results suggest that toludesvenlafaxine can easily pass the blood brain barrier and enter the brain, with highest drug concentrated in the hypothalamus tissue.

In summary, this study characterized the *in vitro* binding affinity and reuptake inhibiting activities of toludesvenlafaxine at DAT, NET and SERT, which confirms its triple reuptake inhibitor profile. In two widely-used rat depression models, toludesvenlafaxine demonstrated dose-dependent and significant antidepressant-like activity with notable effect on preventing testosterone decrease. Toludesvenlafaxine readily enters the brain and, along with its major metabolite ODV, concentrates at specific brain regions such as the hypothalamus. The toxicity studies confirmed toludesvenlafaxine had good safety and tolerability properties in acute, subchronic oral toxicity (13-weeks), and genotoxicity evaluations (Li et al., 2018). fertility and early embryonic development (Guo et al., 2018). No CNS-stimulating (e.g., hyperactivity, irritation or hyperreactivity) or CNS-depressing (e.g., sedation, hypolocomotion, muscle relaxation) effects were observed after acute treatment or repeated dosing treatment with toludesvenlafaxine (Li et al., 2018). Preclinical abuse potential evaluations have been systematically studied which suggest that toludesvenlafaxine has no abuse potential (unpublished results).

Combined, this study for the first time systematically characterized a novel tri-reuptake inhibitor, toludesvenlafaxine, and lays the foundation for future studies to translate preclinical results to therapeutic efficacy in patients with major depressive disorder. Toludesvenlafaxine has finished all preclinical studies and is currently under phase III clinical trial for the treatment of major depressive disorder (clinical trial registration identifier: NCT04853407).

## Data Availability

The original contributions presented in the study are included in the article/supplementary material, further inquiries can be directed to the corresponding author.
